# Nanoparticles for Biomedical Application and Their Synthesis

**DOI:** 10.3390/polym14224961

**Published:** 2022-11-16

**Authors:** Iva Rezić

**Affiliations:** Department of Applied Chemistry, Faculty of Textile Technology, University of Zagreb, 10000 Zagreb, Croatia; iva_rezic@net.hr or iva.rezic@ttf.hr

**Keywords:** biomedical application, nanoparticles, drug delivery, imaging, photo-thermal therapy, sensors

## Abstract

Tremendous developments in nanotechnology have revolutionized the impact of nanoparticles (NPs) in the scientific community and, more recently, in society. Nanomaterials are by their definition materials that have at least one dimension in range of 1 to 100 nm. Nanoparticles are found in many types of different technological and scientific applications and innovations, from delicate electronics to *state-of-the-art* medical treatments. Medicine has recognized the importance of polymer materials coated with NPs and utilizes them widely thanks to their excellent physical, chemical, antibacterial, antimicrobial, and protective properties. Emphasis is given to their biomedical application, as the nanoscale structures are in the range of many biological molecules. Through this, they can achieve many important features such as targeted drug delivery, imaging, photo thermal therapy, and sensors. Moreover, by manipulating in a “nano-scale” range, their characteristic can be modified in order to obtain the desired properties needed in particular biomedical fields, such as electronic, optical, surface plasmon resonance, and physic-chemical features.

## 1. Introduction

The current advancements in the field of biomedical application of nanoparticles is the result of the development of the synthesis and application of the engineered nanoparticles. 

There is a wide variety of polymeric and metallic nanoparticles that are widely explored for possible biomedical applications. By this, such investigation is the focus of extensive research focused on the characterization and modification of intrinsic characteristics, including electronic, optical, and physicochemical properties, as well as surface plasmon resonance. All of the mentioned properties are changed during the modification of particular nanoparticle characteristics, for example, of their size, shape, or aspect ratio. [1].

Nanoparticles can be easily synthesized and modified so that they have novel electronic, optical, magnetic, medical, catalytic, and mechanical properties. Such a powerful modification results in a high surface-to-volume ratio and quantum size effect, which depend greatly on their size, structure, and shape. By deposition of targeted nanoparticles in polymers, new materials foreseen as biomedical devices are made.

Such materials are found in woven and nonwoven medical materials, polymers, and in other applications. The list of NPs applied on textile and polymer materials is presented in Table 1. 

It is estimated that, among all different engineered nanoparticles, silver nanoparticles have the largest degree of commercialization [2]. A great diversity of synthetic bottom-up and top-down methods is used for developing and producing nanoparticles of various chemical composition, size, and shape [3]. However, most of those methods are wet chemical processes based on solution-phase colloidal chemical reactions and often harsh conditions. Except for those widely applied routine procedures, NPs can be produced by enzyme-mediated reactions. 

The production of NPs by enzymes offers many advantages: reactions are performed at ambient temperature, moderate pH values are sufficient for an effective enzymatic reactions, the control of reactions is easy, produced NPs can be easily combined with other organic or heat sensitive materials (e.g., proteins), the crystalline phase of the produced NPs can be different from the one(s) obtained by conventional methods and lead to new products, and the morphology of NPs can be controlled by enzymatic reaction engineering. One of the most important features of the nanoparticles is their small size. It is reported that, by comparing the size of novel engineered nanomaterials with well-known biological nanostructures, such as the DNA double-helix with a diameter of 2 nm or Mycoplasma bacteria with a length of 200 nm, nanoparticles cover this exact region. Through this, it is possible to engineer new colloidal nanoparticles in order to become “*living nanodrugs*” that mimic bacteria. Moreover, “*robotic nanodrugs*” could be designed to manipulate molecular events in human or animal bodies [4]. 

Interaction of NPs with living organisms can be monitored at different levels, from molecular/cellular to higher, tissue systematic levels. Moreover, their interaction will depend on different routes of administration that include inhalation or intranasal, -venous, -dermal, -muscular, and others.

Interactions are under investigation of many interdisciplinary fields and scientists from both a toxicological and therapeutic point of view in the development of a new generation of nano-immuno-therapeutic drugs using in vitro and in vivo preclinical trials. It has to be emphasized, however, that although primary interaction of NPs with immune cells is related to the physical and chemical properties of the NPs, direct conclusions on the mechanisms cannot be foreseen [4,5].

**Figure 1:**
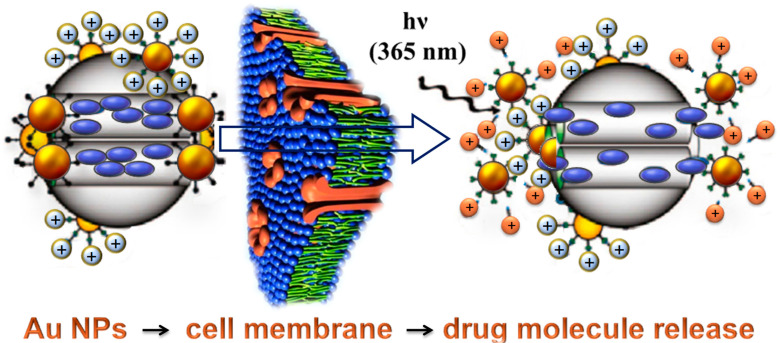
Schematic illustration of the photo-induced intracellular controlled release of gold nanospheres. Upon UV irradiation, the photo-labile linker on the gold NPs is cleaved, changing the surface charge of gold NPs from positive to negative. The charge repulsion between the gold NPs then uncaps the mesopores and allows the release of the drugs for cancer therapy [5].

Man-made polymers are synthesized in a controlled manner and final products contain well-defined characteristics such as defined molar mass, architecture, hydrophobic or hydrophilic properties, crystallinity, and functional groups in the structure of the molecules. Special interest is also given to gold nanoparticles (Au NPs) with or without a polymeric coating. This focus originates from the fact that Au NPs exhibit two very valuable advantages that make them the “stars” among a wide variety of NPs for bio-applications: biocompatibility and ease of surface modifications by a wide range of molecules (Figure 1). Figure 1 presents the illustration of the mechanism in which the photo-induced reactions enables the release of biologically active nanoparticles (gold nanospheres).

The most relevant synthetic methods for producing Au NPs are as follows: seed-mediated method for producing nano-rods and nano-prisms in a two-step process (Zsigmondy nuclear method), thermal decomposition (polyol synthesis) using polyvinylpyrrolidone (PVP) as a surface-capping agent, template mediated synthesis for non-spherical Au NPs in which Au is generated in situ and shaped into a morphology complementary to a template (like channels within porous materials, organic surfactants or block polymer, and biological molecules such as DNA or viruses), and galvanic replacement reaction introduced by Brenner and Riddell comprising the spontaneous reduction of metal ions to metallic particles and films in the absence of an external electric field. In contrast to those methods, enzymatically catalyzed reactions are used to achieve environmentally friendly and cost-effective conditions for the production of NPs. Among a great variety of Au NPs that can be prepared in a controlled way with a high yield and reproducibility, especially important are nanorods, prisms, shells, cages, and hollow nanostructures like stars. Gold shells are spherical NPs consisting of a dielectric core (silica, polystyrene, or sodium sulfide) covered by a thin layer of gold. 

Applications of Au NPs can be categorized into different technological areas (energy, environment, information technologies, and bio-applications) and are based on their outstanding properties [6], such as, for example, optical property to absorb and scatter light with extraordinary efficiency [7] (Genet and Ebbesen, 2007). Recent applications cover the application of Au NPs in photovoltaic devices and environmental conversion of CO into CO_2_, as well as in the design of antennas, lenses, and resonators [5]. Important medical applications of Au NPs today are present in drug delivery [8]. Those are proposed for the use of laser irradiation, pH change, ionic concentration change, and similar reactions that trigger a drug release (Figure 1). Because of their biocompatibility, these NPs can be used with a near-infrared laser source to thermally destroy cancer tissue without significant damage to surrounding healthy tissue. Therefore, gold nanoshells are already in advanced stages of Food and Drug Administration (FDA) clinical trials. Except for drug delivery, medical usage of Au NPs includes medicine diagnosis and therapy, imaging of tumor cells, and other applications listed in Table 2.

Although there is a wide variety of methods for the characterization of NPs in different textile and polymer samples [9], not all of them can reveal the particle size, morphology, and size distribution [2,3]. Among the mostly used methods are scanning electron microscopy (SEM), transmission electron microscopy (TEM), photon correlation spectroscopy (PSC), surface area analysis (BET), and X-ray diffraction (XRF) peak broadening analysis [10]. Characterization methodology is crucial for targeting of special nanoparticles for desired application (Table 2). However, specific targeting using desired ligands can be hard to achieve, and the comparison between targeted and non-targeted NPs obtained similar results [4]. Moreover, the interaction of NPs with biological fluids at the contact point site of drug administration (oral, intranasal, or intravenous) makes the problem even more complex. Some strategies are implemented to manipulate this effect, such as PEG nanoparticles that have the ability to avoid interactions with other molecules. Secondly, there are NPs coated with cell membranes that are able to reduce their blood clearance and, thirdly, there are NPs that are able to drain lymph nodes. All of those properties influence the effectiveness of NPs in reaching their target. More importantly, understanding of the functioning of our immune system provides innovative ideas for the application of immunotherapies through other routes of administration (intraperitoneal injection of NPs or subcutaneous administration of NPs for vaccination purposes) [4]. Furthermore, it has also been shown that certain NP compositions exhibit per se, without the addition of any drug, immunostimulant or immunosuppressive properties [5]. Many examples are provided, from the hyaluronic acid, PLGA, chitosan NPs, or porous silicon micro particles—all of these have shown the ability to act as self-adjuvants or stimulate M1 or Th1 responses.

There are many advantages of nanoparticles. Firstly, the advantages of NPs are their bioactive properties, good attachment to other polymeric materials, not complex mechanisms of formation of composite and bio-composite materials, as well as simple steps in the modification and surface functionalization of different carriers with them. However, a strong hydration shell, precipitation and turbid solution, surface aggregation, dispersive composition, size, and other properties are only a few disadvantages that make them very hard to operate and use in technological and biomedical applications. Moreover, the disadvantages of nanomaterials are the consequence of their small dimensions. Because of this, they easily infiltrate through the cell walls of skin and lung cells. Even worse, they can cross the blood–brain barrier.

## 2. Polymeric Nanoparticles in Biomedical Application

Polymeric nanoparticles (NPs) contain a core with an inner filling that can contain dyes, other inorganic nano-particles, or drug molecules. The outer shell contains usually hydrophilic polymers. Such an outer shell can be created in such a manner that additional functional units are combined with it [10]. 

A biomedical application of polymeric nanoparticles is presented in the following parts of the manuscript.

### 2.1. Polymeric Nanoparticles in the Treatment of Inflammation

The World Health Organization (WHO) has recognized the most abundant chronic diseases of global human population as the following: Alzheimer’s disease, cardiovascular diseases, cancer, chronic obstructive pulmonary disease, and diabetes (type 2). 

Chronic inflammation is recognized as the underlying cause of most chronic diseases, in which the immune system is activated for long periods, resulting in various pro-inflammatory cytokines that induce damage to organisms [4]. Owing to the lack of specificity, conventional therapies that involve non-steroidal and anti-inflammatory drugs have numerous side effects, while at the same time, the application of anti-inflammatory drugs results in low bioavailability. In contrast, specially designed nanocarriers offer possibilities of overcoming such obstacles through the optimization of site-specific delivery and improvements in the solubility of the drugs. This so-called nano-carrier designs is adapted from the microenvironment of the inflamed tissue. The microenvironment has many characteristics, including increased permeability, acidic pH value, and a high presence of reactive oxygen species (ROS) [4,6].

### 2.2. Polymeric Nanoparticles in the Treatment of Cancer

Cancer is the cause of more than 10 million deaths per year, which makes it the second leading cause of death around the world. One of the major drawbacks of conventional medical approaches and therapies is the difficulty in discriminating cancer cells from healthy cells. Nevertheless, there are other distinctive properties of cancer cells, useful in providing new targeting strategies. Firstly, there is an extracellular environment of cancer that is acidic. Secondly, the temperature of the cancer cell seems to be higher than that of the environment. Thirdly, the cancer-associated enzymes and the surface molecules expressed on cancer cells, together with the hypoxic conditions and reductive oxygen species (ROS), are specific properties of such diseases. 

The answer to such a specific regime is polymeric NPs that are able to be easily modified and manipulated to a desired size and surface architecture. Moreover, as the addition to the effect of the passive targeting approach, particles are prepared in range of desired size between 10 and 200 nm. Such a strategy enables improvements in localization and drug delivery uptake in cancer-recognizing molecules for active targeting. 

There is a wide variety of different polymeric NP delivery systems of anticancer drugs investigated for targeting different cancers. Furthermore, the curcumin can be combined with efficient cancer-targeting delivery systems using appropriate biocompatible polymers (PLGA, lecithin, chitosan silk fibroin, and Eudragit^®^). In comparison with free curcumin investigations, the results obtained with curcuma in nano-formulation achieved a huge increase in bioavailability as a result of better adhesion to cancer cells in the colon.

### 2.3. Polymeric Nanoparticles in the Treatment of Infectious Diseases

Microorganisms are more and more resistant to antibiotics. The World Health Organization presented data and outlined this problem as one of the worst threats to public health [11]. 

Various materials that patients in hospitals come into contact with (such as bedding, towels, sheets, bandages, catheters, and other hospital equipment) can present sources of danger if they are not sterile, so there is an imperative that technology aims to develop such materials that will have protective and preventive action against antibiotic-resistant microorganisms [12].

*Staphylococcus aureus* can cause various infections, especially infections on skin, soft tissues, bones, and blood vessels, which is the most common consequence of postoperative surgery. Some strains of *S. aureus* can cause various specific symptoms including toxic shock syndrome. The first strains of resistant microorganisms appeared in the 1960s. Initially, they appeared mainly in hospitals, but over the past decade, the appearance of MRSA has significantly expanded to several countries around the world (WHO 2014, Figure 2). *S. aureus* mortality decreased significantly between 1981 and 2000, but, as incidence rates doubled, the total number of deaths increased, emphasizing the need for further preventive measures and the development of new materials (Benfield et al., 2007). In hospitals, open-wound patients, inhaled devices, and those with impaired immune systems have a higher risk of infections from a wider population. MRSA infections after surgery are rare, but may occur in wounds, chests, or bloodstream (bacteriemia). MRSA infections occur in 1 to 33% of cases in persons undergoing surgery and infections can endanger life and cause a prolongation of hospital stay.

The first wave of strain *S. aureus* resistance to penicillin appeared in the mid-1940s; therefore, since then, *S. aureus* can be divided into strains *resistant (MRSA)* or *sensitive (MSSA)* to methicillin. MRSA infection results in 50% higher probability of death in hospital compared with MSSA infection [14] The most frequent incidence of MRSA infection in hospitals has led to greater use of other antibiotics such as vancomycin, resulting in vancomycin-resistant *S. aureus (VRSA)* microorganisms. The first such case was described in Japan in 1996 [15] and afterwards in England, France, and the United States. Furthermore, *S. aureus* causes many types of infections:

- Skin (McCaig et al., 2006, [16]);
- Food poisoning (Wieneke et al., 1993 [17]);
- Soft tissues, bones (Sheehy et al., 2010 [18]);
- Bacteriemia–blood vessels (Khatib et al., 2009 [19]);
- Infective endocarditis (Fowler et al., 2005 [20]);
- Infections–pneumonie [21].

Untreated bacteriemia of *S. aureus* has a mortality rate of 80% [19,22]; most patients do not survive the first year [23] and, in Western European countries, very often patients in hospitals do not receive adequate therapy at the time [22]. A major problem in hospitals is non-sterile materials, because MRSA is easily transmitted through contaminated hands, clothing, or nesterious medical supplies after contact with a patient infected with MRSA either directly (after contact with a patient, blood, tissue fluid, secretions, and excretions) or indirectly (through contaminated instruments, objects, equipment, surfaces, and similar materials). The spread of MRSA can be prevented by the usage of disposable gloves, capes, and masks, but this is not always feasible. Therefore, new materials need to be developed that are active against microorganisms resistant to antibiotics. In the development of new antimicrobial materials, as well as in the functionalization and modification of existing ones, the application of nanoparticles of metal and metal oxides plays a very important role. The nanoparticles have excellent new medical, mechanical, optical, magnetic, catalytic, and electronic properties owing to a specific surface that is highly dependent on their size, structure, and shape. The global demand for nanoparticles of metal and metal oxide is projected to grow to 1700 tons in 2020 [5].

Nanoparticles have strong antibacterial activity on a wide range of Gram-positive and Gram-negative bacteria, including strains resistant to antibiotics [24]. They have great application because of their chemical stability, catalytic activity, and high conductivity. Rezić et al. have shown that silver nanoparticles have higher antibacterial activity owing to their high surface-to-volume ratio, which ensures better contact with microorganisms [4]. Furthermore, Al-Dhabi et al. have demonstrated visible antimicrobial activity against pathogenic wound infections such as *Bacillus subtilis*, *Enterococcus faecalis*, *Staphylococcus epidermidis*, multidrug-resistant *Staphylococcus aureus*, and *Escherichia coli* [25]. Silver nanoparticles with their own antimicrobial activity in combination with antibiotics (such as penicillin G, amoxicillin, erythromycin, clindamycin, or vancomycin) enhance the action of antibiotics in the treatment of resistant *Staphylococcus aureus* and *Escherichia coli* infections [26].

The interaction between nanoparticles and microorganisms is complex. Two interrelated mechanisms are described in the literature: (1) membrane potential disturbance and (2) production of reactive oxygen species (ROS), where nano-particles act as nano-catalysts [27,28]. Microbial membrane damage occurs when nanoparticles electrostatically bind to the surface of the bacterium, resulting in changes in the membrane wall and membrane potential and loss of integrity. Reactive oxygen species (ROS) are the cytotoxicity of nanoparticles in vivo and in vitro conditions where oxidative stress on the surface damages the integrity of microbial membranes by lipid peroxidation, further damaging the protein and enzyme function and damaging DNA and RNA. In some cases, ROS is induced by either visible or UV light and photocatalytic nanoparticle toxicity, such as, for example, TiO_2_ nanoparticles, which, under UV light, cause lipid peroxidation, respiratory dysfunction, and death of methicillin-resistant *Staphylococcus aureus* cells [29]. Other mechanisms of toxicity of bacterial cell nanoparticles include direct inhibition of specific essential enzymes, induction of nitrogen reactive species [30], and induction of programmed cell death or apoptosis [31]. Table 3 shows the activity of silver nanoparticles of various dimensions on microorganisms.

Polymers containing metal nanoparticles and metal oxides have antimicrobial effects [32], but their effects on many pathogenic microorganisms have not yet been sufficiently explored. The main antibiotic groups currently used affect three bacterial targets: cell membrane synthesis, translation, and DNA replication [33]. Unfortunately, bacterial resistance can occur on each of these antimicrobial effects. The main mode of nanoparticle effect is direct contact with the bacterial membrane, without the need for penetration of the membrane and the wall, which opens the possibility for the microorganisms to grow more resistant to them. Today, silver nanoparticles are used in coatings on many materials such as surgical instruments, catheters or masks, orthopedics and dentistry (as additives for bone and dental materials), diagnostics (for increasing sensitivity of bio-detection), and ultra-susceptible clinical tests for the diagnosis of infarction myocardium and fluorescence detection of RNA [34] (Table 4).

In addition to the pharmaceutical industry, nanoparticles of silver are used in the cosmetic industry thanks to their strong antibacterial and anti-inflammatory properties, in cosmetic products such as deodorants, anti-aging creams, and so on. Nanotechnology can solve the problem of bio-film formation by using an antibacterial active surface with a combination of ZnO and MgO nanoparticles that is activated in the dark and is effective against *MRSA* species [36]. 

Considering the new solutions, it is not surprising that resistance to nanoparticles by microorganisms has already occurred; Panaček et al. demonstrated that gram-negative bacteria *of Escherichia coli 013*, *Pseudomonas aeruginosa CCM 3955* and *E. coli CCM 3954* can develop resistance to silver nanoparticles after repeated exposure and resistance results from the production of adhesive flagellin protein, which activates the aggregation of nanoparticles [37].

Research on antimicrobial nanoparticle coatings has also been applied in the space program: recently, the *International Space Station* tested a silver and ruthenium coating that showed excellent properties against Gram-negative and Gram-positive bacterial strains, including pathogenic bacteria with multiple MRSA resistance, *Enterococcus faecalis*, *Staphylococcus epidermidis*, *E. coli* pathogens (ESTEC), *Pseudomonas aeruginosa*, *Acinetobacter baumannii*, and *Legionella*. The International Space Station is an extremely closed area where bacteria develop special antibiotic defense mechanisms by developing a thicker cell wall or highly express virulent genes. Moreover, the microgravity and cosmic radiation can increase the virulence of microorganisms and transform them into potential pathogens. In addition, these conditions also reduce the immune defense of astronauts, which, when linked to psychological stress associated with space flights, makes them much more prone to infections [38].

### 2.4. Polymeric Nanoparticles in Implants and Prosthetic Devices

One of the most common uses of NPs is in implantable devices. Implantable devices must possess specific requirements, including good biocompatibility, tissue affinity, corrosion resistance and particularly antibacterial property. There is a wide variety of different classical medical devices, implants, and prosthetics: catheters, dental implants, pacemakers, prostheses, and others. Such materials come in direct and prolonged contact with biological tissue. Therefore, their biocompatibility is a critical property that limits their application. 

Frequently reported problems are related to toxicity, allergic reactions, inflammation, and sometimes bacterial infections [39].

### 2.5. Polymeric Nano-Particles as Theranostic Devices 

Therapeutics systems simultaneously act as therapeutic and a diagnostic agent. They are designed to enable target-specific drug delivery while at the same time monitoring the release of the drugs. Moreover, they can monitor the progression of the disease progression while at the same time delivering the treatment response. Although traditional diagnostic imaging (magnetic resonance imaging, computed tomography, and angiography) operates with inorganic contrast agents, organic fluorophores represent a promising alternative, as they show a good safety profile. 

### 2.6. Other Applications 

There are many other applications, from formulations created for skin, hair, nails, or for other formulations. For other applications, there are different sizes of nanoparticles (from the range of 50 to 500 nm), as those enable the penetration through the skin into the organism. Through this property, the development of so-called “transdermal drugs” was enabled [39]. Polymers used as carriers are chitosan, albumin, PLGA, or PLA. 

In addition, inorganic nanoparticles are useful in antibacterial coating; thus, for example, TiO_2_ coated with silver-doped hydroxyapatite or silver-coated collagen proved to possess antibacterial activity useful for catheters and dental implants effective against *S. aureus* and *P. aeruginosa* [40]. 

## 3. Synthesis of Nanoparticles for Biomedical Application

The first attempt of synthesis of metallic particles by enzymes was performed by McConnel and Frajola (1961), when the synthesis of carbonate apatite was carried out in the presence of carbon anhydrase. Today, the advents and developments in nanotechnology resulted in a wide range of microorganisms and their enzymes, which can be utilized in the production of NPs. 

There are three chemical synthetic pathways for synthesis of nanoparticles: the Turkevich method, Brust–Schiffrin method, and seed growth method.

John Turkevich reported his method in 1951 using Na-citrate as a reducing agent and stabilizer for creating spheric NPs of size of 10 to 20 nm. Similarly, the Brust–Schiffrin method from 1994 enabled formation of 1.5–5.2 nm NPs, using thiole at an ambient condition. Lastly, the seed growth method enabled excellent control of size and shape. 

In contrast to conventional chemical synthetic pathways, there are many green methods that use plant extracts or microorganisms in the synthesis of NPs. New methods are developed in order to avoid application and huge usage of unwanted toxic chemicals that are usually used in synthesis of NPs. Those approaches are known as *green methods* in which plant extracts and microorganisms are used for the synthesis of AuNPs as substitutions for toxic reagents. 

Although a reduction step is also involved in this process, this methodology is known as green synthesis owing to the utilization of herbal extracts. Another advantage of this procedure is the fact that NPs prepared using this methodology do not require a further functionalization agent [39]. 

Some examples of green synthesis of biomedically applicable nanoparticles are presented in Table 5.

There are several biopolymers used in the synthesis of nanoparticles: PVP, PEG, poly- vinyl alcohol/, -/acryl amide, -/ally amine, -/phenols, and -/methyl amino ethyl metha crylate. In addition, different surfactants can be used in this reaction [39].

The NPs can also be prepared using biopolymers. Such a procedure can be performed using a single biopolymer or their combination with other reducing agents.

Some of the useful biopolymers are as follows: pectin, glucose, dextrin, amino-dextrin, gum Arabic, starch, chitosan, proteins, gelatin, collagen, casein, tyrosine, alpha-amylase, aspartic acid, tyrosine, tryptophan, glutamic acid, sodium glutamate, cystine, and L- leucine, among others [39] The organic reagents used are as follows: amino alcohols, glycerol, luminol, nitriloacetic acid, and sodium rhodizonate, enabling the reduction of gold NPs from HAuCl_4_.

Enzymatic synthesis of nanoparticles at moderate temperatures will be investigated in this project, because it is of prime interest for the industry; that is, mild manufacturing conditions will ensure low energy consumption, economical production, less manpower, and compatibility with other fabrication processes. Combinations of NPs with proteins allow medical usage of NPs as therapies for effective cancer treatment. Moreover, if the synthesis of NPs is carried out in enzyme-mediated reactions, chemical and physical properties of products can differ from the properties of NPs produced without the presence of enzymes, providing wide variety of their possible novel applications. By understanding this mechanism, the reactions can be guided in the direction of production of targeted nanoparticles with desired applicable properties. 

Enzymes are protein catalysts that are characterized by having a high degree of specificity for their substrates. They catalyze specific chemical reactions: hydrolysis, reduction-oxidation, phase transition, elimination of specific functional groups, and isomerisation. Although the enzymatic reactions naturally occur in living organisms, thanks to technological development, they can now be utilized in bioreactors in different scientific and industrial laboratories with the goal of producing different chemical compounds (drugs and detergent products), as well as nanoparticles [45]. 

*Urease* is a very interesting enzyme for producing NPs. It can be isolated from different microorganisms (*Aspergillus niger*, *Proteus mirabilis*, *Bacillus subtilis*, and *Aerobacter aerogenes*) and applied for the production of NPs of different morphologies like thin films, hollow micro- and nano-spheres, nanotubes, 2D patterns, 3D replicas of biominerals, and others [43]. Therefore, the *urease*-mediated synthesis of NPs is an attractive research field that enables the possibilities of producing NPs for a wide variety of applications: in cancer therapy, for producing materials of special purposes, in food packing, geotextiles, and many others [46]. 

Methods for producing NPs by *urease*-catalyzed reactions can be divided into two main categories: (i) reactions in which the precipitants of metal ions are produced by enzymatic reactions (metal ions in solutions precipitate into oxides, hydroxides, carbonates, or phosphates), and (ii) reactions in which enzymes directly interact with metal-containing substrates to produce metallic materials [42]. 

Reactions using *urease* are usually very simple and obtained NPs can be formed as hydroxides, carbonates, hydratized oxides, or other chemical species. For example, if a small amount of *urease* is diluted in a solution containing calcium chloride and urea, the *urease* will hydrolyze urea into ammonia (carbonic acid will also be produced). 

In the same reaction, calcium ions react with the carbonate at room temperature, and calcium carbonate is produced [47,48]:

H_2_NCONH_2_ + 2 H_2_O → 2 NH_3_ + H_2_CO_3_ (1)

Ca^2+^ + CO_3_^2−^ → CaCO_3_ (2)

The crystallinity, size, and mechanism of growth of produced NPs are governed by reaction parameters (e.g., concentrations, stirring, pH, temperature, pressure, and ultrasound effects). In this project, the kinetic of such enzymatically catalyzed reactions will be thoroughly investigated by different classical instrumental methods. In addition, *beyond-state-of-the-art* analytical and bioanalytical methods will be implemented for revealing the mechanisms of preselected reactions. Unuma et al. (2004) applied *urease* immobilized on alginate gel particles to produce porous aluminum NPs [43]. 

In this reaction, after reaction (1), the following reaction occurred:

Al^3+^ + 3NH_3_ + 3H_2_O → Al(OH)_3_ + 3 NH_4_^+^ (3)

Under high temperatures, aluminum hydroxide produced in reaction (3) converts into Al_2_O_3_ with a particle size of 1–2 mm (Unuma et al., 2004). Porous NPs of iron oxide are produced by a similar reaction:

Fe^3+^ + *x*OH^−^ + *n*H_2_O → FeO_y/2_(OH)_3 − y_ × *m*H_2_O (4)

**Figure 3:**
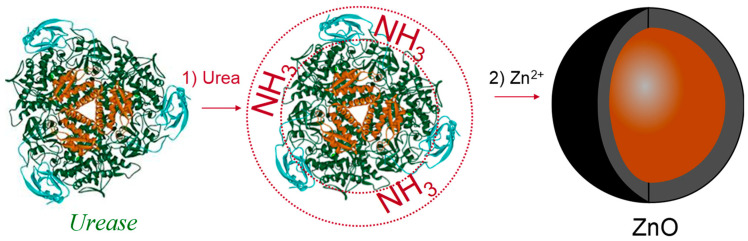
Synthesis of ZnO nanoshells using the *urease* enzyme as a catalytic template. In the first step, NH_3_ is generated by the hydrolysis of urea. This adjusts the pH value around the enzyme. In the second step, Zn^2+^ is added. The growth of nanoshells is governed by the local pH value at the enzyme/solution interface (De la Rica and Matsui, 2008).

In contrast to the process of producing aluminum NPs (reaction 3), process (4) also utilizes the polymer matrices molecules, which degrade after drying and calcification in order to obtain porous iron NP structures [49]. In the reaction of *urease* with ammonia and carbonates, many other metallic NPs can be produced: magnetite [50], iron hydroxide [51], aluminum sulfate [52], calcium carbonate [47,53], strontium and barium carbonate [48], calcium phosphate [54], yttrium hydroxide [55], magnesium oxide, and zinc oxide, in the form of nano-shells (Figure 3).

Targeted products’ metallic NPs have biological and medical activity, antimicrobial, water-repellent, and other protective and polymer-applicable properties. Owing to the special characteristics of the mechanism of its reaction, catalytic *urease* processes can be used for the synthesis of Al, Au, Ca, Cr, Fe, Ge, Pd, Pt, Ti, Y, Zn, and Zr nanoparticles. Some of those NPs (Ca, Ge, Pd, and Pt) are currently not applied in medical materials, while others (Zn and Zr) are applied only as oxides (Table 1). The produced NPs are subsequently deposited onto a polymer substrate in the form of a thin film. The application of such a coated protective material is foreseen for medical applications.

*Urease* is not the only enzyme that can be used. In addition, four oxidoreductases can be utilized: *cellobiose dehydrogenase* (CDH), *glucose dehydrogenase*, *glucose oxidase*, and *laccase*. These enzymes have many applications in biotechnology and are used for producing metallic NPs. For example, *glucose oxidase* extracted from *Aspergillus niger* can be used for production of different nanoparticles (e.g., Au, Sn, and others) [53]. The usage of CDH for producing gold nanoparticles has already been investigated and reported in the literature [54]. Their results showed that CDH acts as an electron donor and reduces gold from [AuCl_4_]^−^ complex into metallic gold nanoparticle by direct electron transfer (DET) at pH 5 or by mediated electron transfer (MET) at pH 7. The limiting factor for NPs’ growth is the ability of CDH to interact with the growing Au NPs. This is an important fact, useful for controlling the direction of the reaction. For example, the ability of enzymes to control the rate of nucleation of NPs is the key step in using them as plasmatic nano-sensors. Having all of this in mind, Au NPs should be the first targeted metal ion for biomedical applications [56,57].

## 4. Characterization of Biomedical Active Nano-Particles

There are many different methods for revealing the mechanisms of metallic NPs’ biomedical application: “*beyond-state-of-the-art*” instrumental methods such as gas-phase electrophoretic mobility molecular analyzer (GEMMA) [58,59,60,61], parallel differential mobility analyzer (PDMA) [62], and liquid chromatography/capillary electrophoresis coupled to MALDI and ESI mass spectrometry (LC/CE-MS/MS), as well as classical spectroscopic methods (graphite furnace atomic absorption spectrometry (GF-AAS), inductively coupled plasma optical emission spectrometry (ICP-OES) [63,64,65,66], scanning electron microscopy equipped with the EDX detector (SEM-EDX) [67,68], thermal gravimetric analysis (TGA), Fourier transform infrared spectroscopy (FTIR), UV/VIS spectroscopy, and liquid chromatography (HPLC)), which is convenient to provide important information on mechanisms of reactions and obtaining products of desired crystallinity, size, and shape [69,70,71,72]. The identification of the newly isolated enzymes can be performed by MALDI-TOF/TOF-MS and LC-MS/MS methods. 

Special focus and attention should be given to the determination of the kinetics of the systems studied, as well as to the revealing of the mechanisms of reaction by “*beyond-state-of-the-art-techniques*” GEMMA and PDMA coupled to capillary electrophoresis (CE), which were invented and developed by prof. Allmaier et al., for the analysis of nanosize materials. To the best of our knowledge, the application of GEMMA and PDMA to monitor enzymatic in situ reactions and to follow both of the enzymes and produce nanoparticles, i.e., the size-separated nanoparticles are collected and can be investigated by other techniques as SEM or AFM, is a new, *beyond-state-of-the-art* concept in the field. 

Although different *state-of-the-art* techniques can be today utilized for the in situ measurement of nanoparticles [73,74,75,76,77], such attempts cannot provide insight into the particle growth. In future work, on the other hand, the focus should be shifted to understanding the mechanism of NPs’ growth during the reaction by monitoring reactants and products (enzymes and occurred metallic nanoparticles). Having the possibility to obtain a deep insight into the mechanism of enzymatically guided reactions is crucial, as contradictory observations and conclusions are currently reported in highly ranked scientific journals (*Nature* and *Angewandte Chemie*). For example, B. Sharma et al. (2013) concluded that, during *urease*-guided reaction of the formation of hollow ZnO nanoparticles, the enzyme covers the outer shell (Sherma et al., 2013). This conclusion is in contradiction to the interpretation of De la Rica and Matsui (2008), who reported that the enzyme (Figure 3) fulfills the nanoparticles’ inner core [78]. 

**Figure 4:**
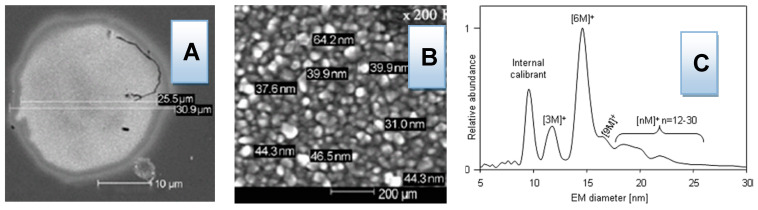
Results of investigation into biomedically active NPs: (**A**,**B**) SEM-magnified images of gold NPs obtained by *cellobiose dehydrogenase* treatment of [AuCl_4_]^−^ (Malel et al., 2010); (**C**) GEMMA spectrum of non-covalent homo-complex of *Jackbean urease* (Allmaier et al., 2008).

Widely used NPs include oxides of Al, Sb, Bi, Ce, Cu, In/Sn, Fe, Mg, Mn, Ni, Si, Ti, Y, Zn, and Zr, thanks to their novel electronic, optical, magnetic, medical, catalytic, and mechanical properties resulting from the high surface-to-volume ratio and quantum size effect. The primary target of biomedical application research should be to modify and control protective materials using newly prepared NPs (e.g., Ag, Al, Au, Cr, Ge, Fe, Pd, Pt, Ti, Y, Zn, and Zr NPs). Polymer materials coated with thin films of NPs have special protective properties, such as preventing oxygen, moisture, and microorganism permeability. The process of thin film deposition is very important, as it influences graininess and the structure of the deposit. In other words, the deposit can comprise grains of different sizes and its structure may suffer from defects in connectivity [79]. There are several thin film deposition technologies that can be applied: sputtering, sol–gel deposition, spray coating, chemical vapor deposition, spray pirolysis, cathode electro deposition, anodic conversion, and conventional evaporation. The properties of thin metal films with nano-holes smaller than the light wavelength have recently attracted large amounts of interest, owing to the remarkable observation of extraordinary light transmission through metallic sub-wavelength hole arrays, which does not obey classical optical theory [80]. Therefore, several methodologies should be tested including the sol–gel process of dip coating [81,82,83,84], for combining different NPs and different polymer materials [85,86], in order to create metal NP films of different thicknesses that are most efficient for biomedical applications (Figure 4) [87].

The important nanoparticles in biomedical applications are inorganic and organic nanoparticles. Among inorganic species, gold nanoparticles and silver NPs are today the most investigated and prominent species. 

The advantages of nanoparticles that are important in biomedical application, as well as their synthesis, have been described in this work. However, it has to be emphasized that such nanomaterials can be very toxic. The level of their toxicity will depend on their physical, chemical, and mechanical (morphological) properties. At this point, toxic effects of nanoparticles should be considered before the determination of the most appropriate synthesizing pathway for producing nanoparticles. By optimizing this step, the desired size, shape, composition, and other most important parameters should be varied in order to prove that the result will not have any adverse toxic effects.

Therefore, during the synthesis of NPs by engineering the desired outcome properties, the toxicity can be avoided and minimized. For example, the parameters such as the size and surface area, as well as their shape, aspect ratio, surface coating, crystalline, and dissolution, as well as agglomeration, are dominant parameters. Such parameters will influence the capability of NPs to begin the formation of reactive species (ROS) and all other mechanisms important in cytotoxicity, genotoxicity, and neurotoxicity processes.

There are some identified toxic mechanisms. Those are detected through the induction of the formation of reactive species and cytotoxicity to cells, as well as genotoxic and neurotoxic effects. For example, smaller nanoparticles had a higher rate of acute toxicity in in vivo experiments. In contrast to this field, the area of investigation of polymeric nano-sized materials is much broader and enables a huge variety of different targeting materials. 

However, the advantages of nanomaterials also provide interesting applications in technological and industrial areas. What are the future aspects of the nanomaterials? Firstly, there are carbon nanotubes, which are light materials with special physical properties, because of which they can revolutionize the design of cars by enabling the possibility to conduct electricity and heat. Secondly, there are nano-robots. It is likely that such devices are the next stage in miniaturization and, as such, they can lead to the production of nano-robots that can help cells of living organisms in their daily tasks [88]. Last, but not least, many authors describe nano and micro vehicles that can be operated, moved, and guided to move on particular surfaces; this application is important in many different aspects of life. 

## 5. Conclusions

The field of nanomaterial science is growing so rapidly that it has already resulted in the implementation of materials with nanoparticles in many different innovations. It can be estimated that the application of biomedically active NPs in combination with different polymers in the form of thin films, nanocapsules, or some other form will result in producing new materials with changed physical, chemical, and mechanical properties. For example, properties such as anti-microbial activity, water repellence, and low oxygen permeability can be exploited for biomedically active surfaces. On the other hand, hollow NPs that can release encapsulated molecules can be used in targeted drug delivery. In addition to this, polymers coated with NPs could be beneficial for catheters, prostatic materials, and others. Although there are many review articles with similar topics, many similar articles are focused on only one nanoparticle [89,90,91,92,93,94,95,96,97,98,99,100]. In contrast to the work that can be found in the recent literature, this manuscript covered many different nanoparticles, from inorganic silver, gold, to organic (polymeric) species. More importantly, the most important aspect of this work is the fact that not only the possible biomedical application of nanoparticles is presented, but also their toxicological effect. As this can be minimized by influencing the parameters of the synthesis of such materials, only an integral approach can bring benefits to the desired nanomaterials and their real application.

However, the application of newly created biomedically active materials will depend on their toxicity, environmental impact, and costs of the final product. Significant transfer of knowledge among interdisciplinary researchers is expected in the following years, which should lead to solving particular problems in analytical chemistry, material sciences, and nanotechnology related to biomedically active NPs.

## Figures and Tables

**Figure 2:**
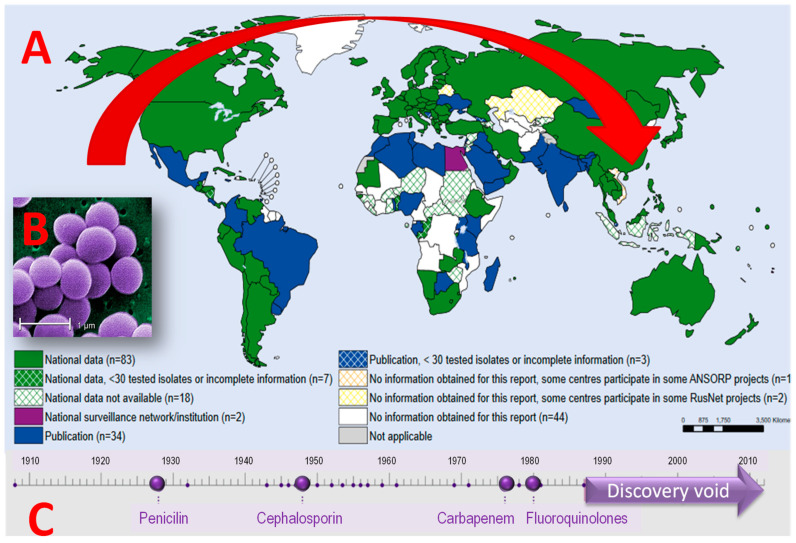
(**A**) Collected data on the number of infections by *S. Aureus* (MRSA), WHO 2014; (**B**) SEM microphotographs of *Staphylococcus aureus* [13]; (**C**) discovery void in new antibacterial drugs during the last 30 years.

**Table 1:** Nanoparticles in biomedical applications [2,3].

NPs	Diameter	NPs	Diameter	NPs	Diameter
Ag	1.5–350 um	Eu_2_O_3_	30–58 nm	Pr_6_O_11_	15–30 nm
Al	18 or 80 nm	Fe	25–250 nm	Si	30–70 nm
Au	50–150 nm	Gd_2_O_3_	15–80 nm	SiO_2_	15–80 nm
B_2_O_3_	40–80 nm	In_2_O_3_	30–50 nm	Sm_2_O_3_	15–55 nm
BaSO_4_	1–5 um	In(OH)_3_	20–70 nm	SnO_2_	45–60 nm
C	3–400 nm	La_2_O_3_	15–30 nm	SrTiO_3_	100 nm
CeO_2_	15–105 nm	Li_4_Ti_5_O_12_	20–60 nm	Ti	30–50 nm
Co	28 nm	MgO	20–100 nm	W	50 nm
Cr	50 nm	Mg(OH)_2_	15 nm	Y_2_O_3_	20–40 nm
Cu	25–500 nm	Mn_2_O_3_	30–60 nm	YbF_3_	40–80 nm
Dy_2_O_3_	30 or 55 nm	Mo	70 nm	Zn	80–130 nm
Er_2_O_3_	20–53 nm	Ni	20–50 nm	ZrC	30–60 nm

**Table 2:** Imaging and biomedical capabilities of Au NPs [5].

	Modality	Nanoparticle/Agent
Imaging	Optical scattering	Au nanoshells, nanorodes, nanocages
	PET, SPECT	Radioisotope ^198^Au
	CT	Au NPs
Therapeutic actuation	Phototermal	Au nanoshells, nanocages
	Photoacoustic	NIR-absorbing Au NPs
	Chemotherapy	Au NPs loaded with anticancer drugs
	Gene therapy	Au NPs loaded with RNA, DNA

**Table 3:** Antimicrobial activity of silver nanoparticles on microorganisms [3,9,32].

Biological System	Effect	Size, nm	Biological System	Effect	Size, nm
*Acinetobacter baumannii*	Antibacterial	5–30	*Micrococcus luteus*	Growth inhibition	~20–~30
*Acinetobacter*	Growth inhibition	~20–100	*multidrug resistant Pseudomonas aeruginosa*	Inhibition of biofilm formation	20–30
*Aeromonas*	Growth inhibition	~20–100	*multidrug-resistant Escherichia coli*	Antibacterial	~6
*Aspergillus foetidus*	Antifungal	104.9	*multidrug-resistant Klebsiella pneumoniae*	Antibacterial	~6
*Aspergillus fumigatus*	Antifungal	5–30	*Staphylococcus aureus*	Bactericide	~10–~100
*Aspergillus niger*	Antifungal	4.75–8.31	*Paenibacillus koreensis*	Antibacterial	~10
*Aspergillus oryzae*	Antifungal	104.9	*Penicillium*	Antifungal	>42
*Aspergillus*	Antifungal	7–20	*Peptostreptococcus*	Antibacterial	~100
*Aspergillus terreus*	Antifungal	Nije specificirano	*Pichia pastoris*	Antifungal	9–10
*Bacillus cereus*	Antibacterial	120	*Propionibacterium acnes*	Antibacterial	20–70
*Bacillus megaterium*	Antibacterial	24	*Proteus mirabilis*	Cytotoxic	Not specified
*Bacillus mycoides*	Bactericide	16	*Proteus vulgaricus*	Antibacterial	50–70
*Bacillus pumulis*	Bactericide	Not specified	*Pseudomonas aeruginosa*	Antibacterial	1–12
*Bifidobacterium*	Antibacterial	~100	*Rhizoctonia bataticola*	Antifungal	~2–40
*Bordetella pertussis Candida albicans*	Growth inhibition	~20–~30	*Saccharomyces*	Antifungal	~15
*Candida albicans ATCC 10239*	Antifungal	~20–45	*Salmonella paratyphi*	Antibacterial	63–90
*Candida glabrata ATCC90030*	Antifungal	Not specified	*Scedosporium JAS1*	Antifungal	Not specified
*Candida tropicalis*	Antifungal	Not specified	*Serratia marcescens*	Antibacterial	Not specified
*Citrobacter*	Growth inhibition	~20–100	*Shigella flexneri MTCC 1475*	Antibacterial	17–29
*Cryptococcus neoformans*	Antifungal	50–70	*Staphylococcus aureus*	Antibacterial	1–12
*Eschericia coli MTCC 443*	Bactericide	8–12	*Staphylococcus aureus ATCC 25923*	Antibacterial	5–25
*Enterobacter aerogenes*	Antibacterial	8–12	*Staphylococcus aureus ATCC BAA-1721*	Antibacterial	~89
*Enterococcus faecalis*	Antibacterial	~89	*Staphylococcus epidermidis*	Antibacterial	20–70
*Fusarium oxysporum*	Antifungal	104.9	*Streptococcus*	Antibacterial	15
*Klebsiella pneumoniae*	Antibacterial	~6	*Trichopyton rubrum*	Antifungal	~15
*Lactobacillus acidophilus*	Bactericide	16	*Vibrio cholerae*	Antibacterial	5–25
*Listeria monocytogenes*	Antibacterial	17–29	*Xanthomonas campestris*	Antibacterial	~54
*MRSA*	Growth inhibition	198–595	*Yersinia enterocolitica*	Antibacterial	-

**Table 4:** Application of silver nanoparticles in medicine items [35].

Area	Application of Silver Nanoparticles
Anesteziology	Coating on breathing masks, endotracheal tubes for mechanical ventilation assistance
Cardiology	Coating on the tracking catheter
Stomatology	Adhesives in dental materials, silver-filled SiO_2_ nanocomposite resins
Diagnostics	Ultra-sensitive and ultra-fast platform for clinical tests for myocardial infarction diagnosis, fluorescence detection of RNA
Drug release	Remote laser induced opening microcapsules
Eye care	Contrasts on contact lenses
Visualisation	Nomenclatures for marking cells
Neurosurgery	Coating of the catheter for the drainage of cerebrospinal fluid
Orthopedics	Bone cement additives, joint replacement implants, orthopedic socks
Drugs	Treatments for dermatitis, ulcerative colitis and acne, HIV-1 inhibition
Surgery	Coats in medical textiles—surgical suits and masks
Urology	Plastered on surgical nets for pelvic reconstruction

**Table 5:** Nanoparticles synthesized by microorganisms [41,42,43,44].

Microorganisms	Nanoparticles	Temperature, °C	Size (nm)	Shape
*Rhodococcus species*	Au	37	5–15	sphere
*Shewanella oneidensis*	Au	30	12 ± 5	sphere
*Plectonemaboryanum*	Au	25–100	<10–25	cube
*Plectonema boryanum*	Au	25	10–600	octahedral
*Escherichia coli*	Au	37	20–30	triangle
*Yarrowia lipolytica*	Au	30	15	triangle
*Rhodopseudomonas capsulate*	Au	30	10–20	sphere
*Brevibacterium casei*	Au, Ag	37	10–50	sphere
*Trichoderma viride*	Ag	27	5–40	sphere
*Phaenerochaete chrysosporium*	Ag	37	50–200	pyramidal
*Bacillus cereus*	Ag	37	4–5	sphere
*Lactobacillus species*	Ba, TiO_3_	25	20–80	tetragonal
*Fusarium oxysporum*	CdSe	10	9–15	sphere
*Escherichia coli*	Cd, Te	37	2.0–3.2	sphere
*Fusariumoxysporum*	CdCO_3,_ PbCO_3_	27	120–200	sphere
*Lactobacillus*	CdS	25–60	4.9 ± 0.2	sphere
*Pyrobaculum islandicum*	Cr, Mn, U, Tc	100	different	sphere
*Shewanella oneidensis*	Fe_3_O_4_	28	40–50	hexagonal
*Enterobacter species*	Hg	30	2–5	sphere
*Desulfovibrio desulfuricans*	Pd	25	50	sphere
*Shewanella algae*	Pt	25	5	-
*Saccharomyces cerevisiae*	Sb_2_O_3_	25–60	2–10	sphere
*Shewanella species*	Se	30	181 ± 40	sphere
*Fusarium oxysporum*	SrCO_3_	27	10–50	needle like
*Fusarium oxysporum*	TiO_2_	300	6–13	sphere
*Fusarium oxysporum*	ZrO_2_	25	3–11	sphere

## Data Availability

Not applicable.
